# Targeting Opposing Immunological Roles of the Junctional Adhesion Molecule-A in Autoimmunity and Cancer

**DOI:** 10.3389/fimmu.2020.602094

**Published:** 2020-11-25

**Authors:** Caio S. Bonilha, Robert A. Benson, James M. Brewer, Paul Garside

**Affiliations:** ^1^ College of Medical, Veterinary and Life Sciences, Institute of Infection, Immunity and Inflammation, University of Glasgow, Glasgow, United Kingdom; ^2^ Research and Development Department, Antibody Analytics Ltd., Newhouse, Lanarkshire, United Kingdom

**Keywords:** autoimmune diseases, cell adhesion, epithelial barrier, F11 receptor, inflammation, junctional adhesion molecule-A

## Abstract

The junctional adhesion molecule-A (JAM-A) is a cell surface adhesion molecule expressed on platelets, epithelial cells, endothelial cells and leukocytes (e. g. monocytes and dendritic cells). JAM-A plays a relevant role in leukocyte trafficking and its therapeutic potential has been studied in several pathological conditions due to its capacity to induce leukocyte migration out of inflamed sites or infiltration into tumor sites. However, disruption of JAM-A pathways may worsen clinical pathology in some cases. As such, the effects of JAM-A manipulation on modulating immune responses in the context of different diseases must be better understood. In this mini-review, we discuss the potential of JAM-A as a therapeutic target, summarizing findings from studies manipulating JAM-A in the context of inflammatory diseases (e.g. autoimmune diseases) and cancer and highlighting described mechanisms.

## Introduction

The junctional adhesion molecule-A (JAM-A), also called junctional adhesion molecule-1 (JAM-1), is a member of the immunoglobulin superfamily and received its first denomination as F11 receptor (F11R), a molecule expressed on the surface of human platelets (1). Only a few years later, this molecule was detected in epithelial and endothelial intercellular tight junctions (2). JAM-A interactions with extracellular ligands assure firm cell-cell adhesion, playing important roles in endothelial cell migration (3–5) and proliferation (6) and epithelial cell barrier functions (7). Changes in barrier integrity caused by disruption of JAM-A pathways can indirectly modulate immune responses by modifying migratory patterns of antigen presenting cells (APC). However, JAM-A is also expressed by immune cells themselves, such as monocytes (2) and dendritic cells (DC) (8, 9). Thus, immune mediated processes are also likely to be directly influenced by JAM-A activity. As our understanding of the contributions of JAM-A to inflammatory process increases so does interest in the therapeutic value in targeting of JAM-A. In this regard, we summarize findings on the disruption of JAM-A pathways in murine models of inflammation and cancer, highlighting possible opposing immunological mechanisms mainly involving leukocyte migration.

## JAM-A Biology

**Figure 1:**
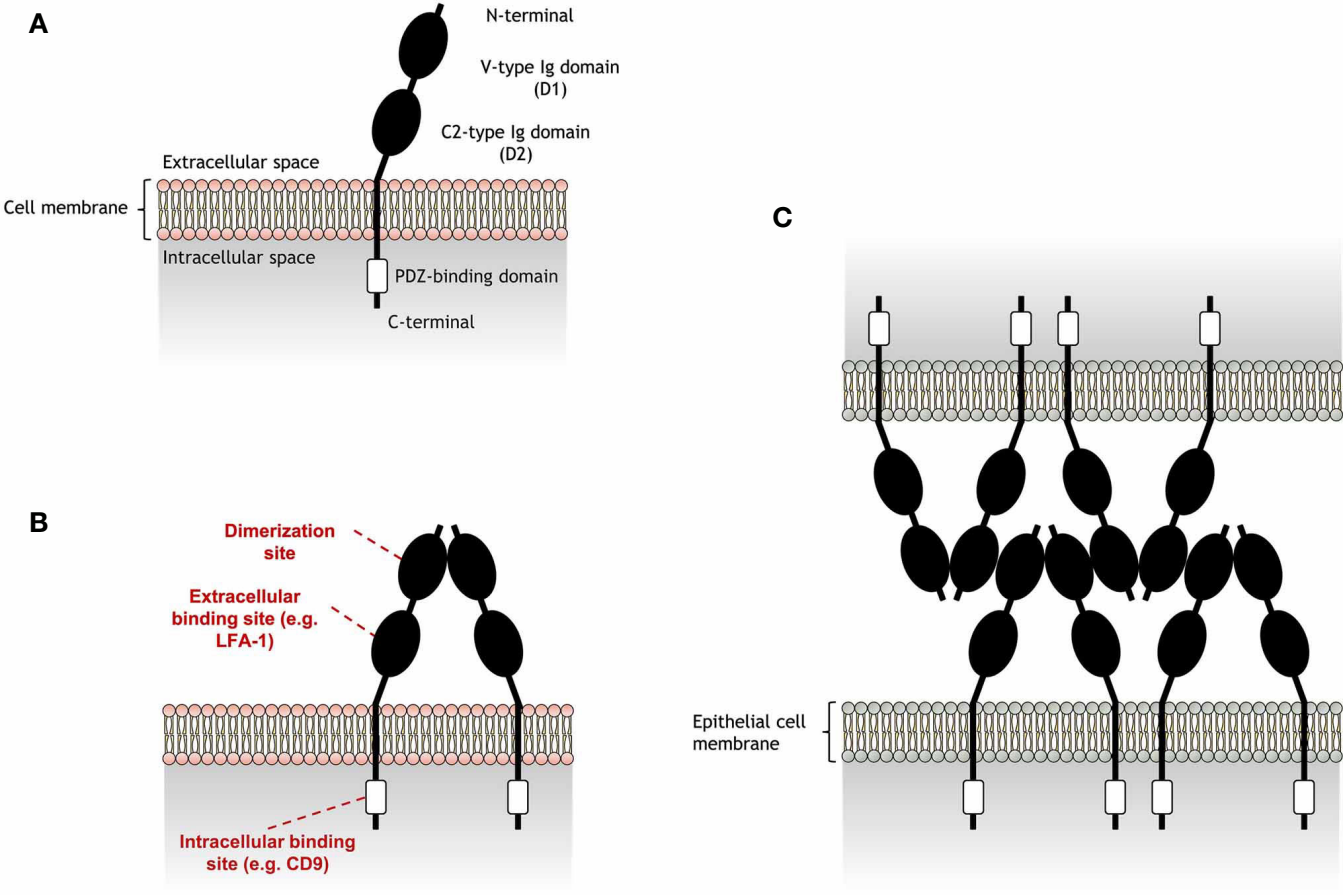
Schematic representation of junctional adhesion molecule-A (JAM-A) structure and homophilic adhesion. **(A)** JAM-A is composed by an extracellular region and a cytoplasmatic tail, connected by a transmembrane portion. JAM-A extracellular portion is formed by a membrane-distal V-type Ig-like domain (D1), which also includes a N-terminal portion, and a membrane-proximal C2-type Ig domain (D2). These Ig-like domains are linked by a short connector region. JAM-A cytoplasmatic tail contains a PDZ-binding motif that is linked to a C-terminal portion. JAM-A can be expressed on the cell surface as monomers, but can also interact with JAM-A monomers in *cis* interactions to form **(B)** homodimers in a process called dimerization. While the D1 domain is the region in which JAM-A monomers interact with each other, the D2 region can bind to other extracellular ligands in *trans* interactions [e.g. lymphocyte function-associated antigen 1 (LFA-1)]. In addition, the PDZ-binding domain allows JAM-A to bind to scaffold proteins, such as CD9, responsible to link JAM-A to β_3_ integrin. **(C)** Endothelial JAM-A homophilic adhesion consists of dimers on opposing cells forming contacts *via* the D1 domain and allows strong cell-cell adhesion in tight junctions for the formation of a molecular barrier that ensures the homeostasis of epithelial barrier integrity. JAM-A *trans* cell-cell arrangement was reported by Kostrewa et al. (10).

JAM-A is a transmembrane glycoprotein composed of a cytoplasmatic tail and an extra-cellular region consisting of a membrane-distal domain (D1) and a membrane-proximal domain (D2) (**Figure 1**). JAM-A homophilic binding (JAM-A-JAM-A) was first suggested to occur in epithelial-epithelial and endothelial-endothelial cell interactions, due to the presence of JAM-A in tight junctions (2). The detection of JAM-A dimers on the surface of JAM-A-transfected epithelial cells strengthened this hypothesis (11), being confirmed by protein- (11, 12) and cell-based (12, 13) assays. Homophilic ligation is mediated by JAM-A D1 domain and allows the formation of JAM-A dimers (14). Dimerization occurs by interaction of JAM-A monomers within the surface of the same cell (*cis* interactions). JAM-A dimers can then interact with other JAM-A dimers (*trans* interactions) to bridge epithelial cells in tight junctions (see **Figure 1C**).

Besides homophilic binding, JAM-A can also undergo heterophilic *cis* or *trans* interactions with other extracellular ligands (**Table 1**). JAM-A can bind to the β_2_ chain of the lymphocyte function-associated antigen 1 (LFA-1), but not of the macrophage-1 antigen (MAC-1), *via* the JAM-A D2 domain (16, 18). The D2 domain was also found to be important in stabilizing homophilic interactions (13). However, the ligation of JAM-A D2 domain to LFA-1 was found to reduce the dynamic strength of JAM-A homophilic interactions in assays with immobilized JAM-A and Jurkat T cells (13), a human cell line expressing considerable levels of JAM-A (16). Immunoprecipitation assays revealed formation of JAM-A and β_3_ integrin (CD61) aggregates in endothelial cell lysates (3), suggesting a direct interaction between these molecules. However, this interaction was later found to be dependent on CD9, as absence of this tetraspanin inhibited JAM-A coimmunoprecipitation with β_3_ integrin (3). Other immunoprecipitation assays suggest that JAM-A can also bind to α_IIb_ integrin (CD41) (15), as well as the β_2_ chain of LFA-1, as previously mentioned. However, CD9 is also known to interact with some of these integrins (19), which may point a dependence on this tetraspanin for the interaction of these integrins with JAM-A. In addition to these ligands, JAM-A can bind to another member of its family, the junctional adhesion molecule-B (JAM-B) (17), and can also work as a receptor for a few strains of murine and human viruses (14, 20–22). While JAM-A *cis* interactions are mainly responsible for cell signaling processes that may indirectly regulate cell migration (4, 15, 23), *trans* interactions directly mediate cell-cell adhesion and are essential for JAM-A role in leukocyte migration.

**Table 1:** Junctional adhesion molecule-A (JAM-A) described extracellular ligands.

Protein	Family	Expression	Detection of interaction	Ligation conformation	References
α_IIb_β_3_ (CD41/CD61)	Integrin	Platelets	Human platelet lysates by co- immunoprecipitation (co-IP)	*Cis*	(15)
α_L_β_2_ (LFA-1 CD11a/CD18),	Integrin	Lymphocytes, dendritic cells, NK cells, neutrophils	JAM-A-transfected Chinese hamster ovary (CHO) cells with immobilized LFA-1 by adhesion assay	*Trans*	(16)
CD9	Tetraspanin	Platelets, endothelial cells, lymphocytes, monocytes, macrophages, dendritic cells, eosinophils, basophils, mast cells	HeLa cell lysates by co-IP Human umbilical vein endothelial cell (HUVEC); lysates by co-IP	*Cis*	(4)
JAM-A (JAM-1 F11R),	Junctional adhesion molecule	Platelets, epithelial cells, endothelial cells, monocytes, dendritic cells	Human platelet with immobilized JAM-A by adhesion assay; JAM-A-transfected CHO cells with immobilized JAM-A by adhesion assay	*Cis/trans*	(12, 13)
JAM-B (JAM-2 VE-JAM),	Junctional adhesion molecule	Endothelial cells	JAM-A- and JAM-B-transfected HEK293T cells by co-IP and proximity ligation assay	*Cis/trans*	(17)

## JAM-A Role in Leukocyte Trafficking

The first report of a role for JAM-A in leukocyte migration comes from Martìn-Padura et al. (2), in which JAM-A blockade was found to inhibit spontaneous and chemokine-induced monocyte transmigration through endothelial cell monolayers. Tumor necrosis factor alpha (TNF-α) and interferon-gamma (IFN-γ)-stimulated endothelial cells redistribute JAM-A on their surface from intercellular junctions to the apical region of the cells, increasing leukocyte adhesion to the inflamed endothelia (16, 24). As such, several other studies have shown endothelial and epithelial cell expression of JAM-A contributes to leukocyte trafficking.

JAM-A blockade was found to inhibit chemokine stimulated neutrophil transmigration across TNF-α and IFN-γ-inflamed endothelium, but not neutrophil arrest (16). Treatment with anti-JAM-A antibody or JAM-A-Fc fusion protein (human JAM-A extracellular domains fused to the Fc portion of human IgG1) inhibited transendothelial migration of human memory (CD45RO+) CD4+ T cells triggered by para-methoxyamphetamine (PMA) and CXC chemokine ligand 12a (CXCL12a) in a LFA-1-depedent manner (16, 25). Splenic CD11c+CD11b-B220+ plasmacytoid DCs (pDC) treated with a different anti-JAM-A mAb also had diminished transmigration through layers of high endothelial venule (HEV) cells, whereas cell adhesion remained unaffected (26). *In vivo*, treatment with anti-JAM-A mAb decreased leukocyte transendothelial migration through cremaster venules induced by IL-1β, but not by chemoattractants, leukotriene B4 (LTB4) or platelet-activating factor (PAF) (27). This same effect was found in JAM-A deficient mice in comparison with wild-type (WT) mice. This reduction in leukocyte transendothelial migration through venules was found to be mediated by JAM-A expressed on endothelial cells, but not on leukocytes.

Although studies in monocytes, neutrophils, memory T cells and pDCs suggest that JAM-A blockade could have an inhibitory effect on leukocyte migration, conventional DCs (cDC) show an increased propensity to cross lymphatic endothelial cells when JAM-A activity is lacking (8). While monocytes, neutrophils (2, 8, 9) and pDCs (26) express very low or undetectable levels of JAM-A, cDCs express high levels of this transmembrane protein (8, 9). Bone marrow-derived DCs (BMDC) treated with anti-JAM-A mAb, or originating from JAM-A knockout (KO) mice, showed increased random motility *in vitro* compared to their respective controls (8). These JAM-A-deficient BMDCs expressed similar levels of maturation markers (CD80 and CD86), surface molecules related to DC migration (CD11a, CD11b, CD11c, CD62L, JAM-B, and JAM-C) and antigen uptake capacity in comparison to WT BMDCs, suggesting that JAM-A may not participate in early stages of DC differentiation and maturation. However, JAM-A deficient BMDCs display increased transmigration across monolayers of lymphatic endothelial cells, but unaffected transmigration across microvascular endothelial cells. *In vivo*, BMDCs from JAM-A deficient mice had increased migration from FITC-painted skin to the LN. The selective transmigration of JAM-A-deleted DCs suggests that DC JAM-A plays a role in homing steps during DC trafficking to the LN from tissues but may not participate in DC tissue infiltration from the vasculature. When analyzing the role of endothelial JAM-A in DC migration, another study reported higher *in vitro* transmigration of JAM-A-expressing BMDCs through layers of JAM-A-deficient lung endothelial cells in comparison with endothelial cells from JAM-A^-/-^ mice reconstituted with full-length JAM-A complementary DNA (cDNA) (28). These studies indicate that while DC JAM-A participates in DC trafficking through the lymphatics, endothelial JAM-A play a dominant role in DC arrest and migration functions through the vascular endothelium. The effects of JAM-A manipulation to the migration of DCs and other immune cells have raised interest in potential JAM-A-targeted therapies for inflammatory diseases and cancer.

## JAM-A Manipulation in Disease Models of Inflammation

Due to its expression in different cell types (platelets, endothelial cells and leukocytes), its capacity to modulate cell adhesion and migration and its upregulation in inflamed tissues, JAM-A has been studied as a therapeutic target in a number of disease models.

### Skin Inflammation

Inflammatory skin sites are characterized by proliferation of dermal cells, dilated blood vessels and accumulation of immune cells (29). In a model of skin inflammation, systemic treatment with an antagonistic anti-JAM-A mAb inhibited leukocyte infiltration upon chemokine administration in subcutaneous air pouches (2). However, in a model of ear skin inflammation driven in an antigen specific manner, JAM-A-deficient mice displayed enhanced contact hypersensitivity (8). The increase in ear swelling in this T-cell mediated model was linked to activity of JAM-A-deficient DCs, most likely arising through their augmented migration to LNs and enhancing activation of antigen specific T cells.

### Vascular Disease

Adhesion molecules have been implicated in vascular wall integrity and play an important role in vascular diseases (30). JAM-A ability to control platelet aggregation (12) have triggered particular interest in this molecule in studies of vascular diseases such as atherosclerosis, a cardiovascular disease caused by the development of plaques on artery walls restricting or blocking blood flow to specific organs or regions of the body (31). In humans, elevated JAM-A gene expression was described in atherosclerotic plaques compared with artery segments of normal patients (32, 33) and in unstable carotid plaques in comparison to stable plaques (33). In addition, JAM-A has been found to be required for human platelet adhesion to inflamed endothelial cells (34).

In models of ischemia-reperfusion (I/R) injury, both JAM-A genetic depletion and blockade with anti-JAM-A mAb suppressed leukocyte infiltration in response to cremaster muscle (27) and liver I/R injury (35). However, no protective effects on microvascular and hepatocellular injury were reported, evidenced by unaffected levels of sinusoidal perfusion and liver enzymes alanine aminotransferase (ALT) and aspartate aminotransferase (AST). The use of mice with JAM-A selective depletion in endothelial cells identified the requirement of endothelial JAM-A in the reduction of T cell, but not neutrophil, transmigration. This dependence on endothelial JAM-A was also reported in a model of heart I/R injury, in which JAM-A absence reduced leukocyte infiltration in the myocardium by affecting transendothelial migration (36).

In mouse models of atherosclerosis, increased *f11r* messenger ribonucleic acid (mRNA) expression was found in both early atherosclerotic endothelium of carotid arteries (25) and advanced atherosclerotic plaques (32). A JAM-A-Fc fusion protein was used in an *ex vivo* perfusion model to demonstrate JAM-A role in early atherosclerosis. Treatment with this fusion protein inhibited the arrest of human monocytes and memory CD4+ T cells in murine atherosclerotic endothelium under blockade of very late antigen 4 (VLA-4), an intercellular adhesion molecule 1 (ICAM-1) ligand (25). These results show the capacity of JAM-A targeting to modulate the recruitment of leukocytes to atherosclerotic endothelium by blocking pathways of competitor ligands.

*In vitro*, treatment with a JAM-A antagonistic peptide capable of blocking homophilic binding (peptide 4D), decreased platelet adhesion to inflamed endothelial cells, whereas agonistic reagents promoted platelet aggregation (12, 37). This antagonistic peptide also decreased plaque number and size and increased survival of mice in an atherosclerosis model in comparison with mice treated with a scrambled peptide (38), due to a reduction on platelet adhesion to the inflamed endothelium. However, in a murine model of thrombosis, lack of JAM-A resulted in increased thrombus formation due to enhanced aggregation of platelets (39). JAM-A^-/-^ mice also had shorter tail-bleeding time and faster vessel occlusion. Although no studies have evaluated the capacity of the antagonistic peptide to block other JAM-A pathways, these studies suggest that, while JAM-A homophilic binding by platelets may be a useful target for avoiding formation of atheroma, other JAM-A pathways may have platelet stimulatory functions and promote blood coagulation that can obstruct and impair the homeostasis of the circulatory system.

### Inflammatory Bowel Disease

The intestinal mucosa forms an important barrier against potential hostile microorganisms and other foreign antigens. The permeability of the intestinal epithelium is mediated by tight junctions, formed of molecular components, such as adhesion molecules, that link intestinal epithelial cells and exert a control on the passage of environmental molecules. Intestinal barrier defects have been associated with chronic mucosal inflammation and inflammatory bowel diseases (IBDs) (40). A few studies have addressed the role of JAM-A in the regulation of intestinal barrier functions during IBDs. Although JAM-A deficient mice displayed higher susceptibility for development of dextran sulfate sodium (DSS)-induced colitis in comparison with WT mice (41), JAM-A depletion increased epithelial proliferation, which resulted in faster repair of epithelial defects, evidenced by a reduction in damaged colonic mucosa. The increase in clinical disease was attributed to a higher permeability in the colonic mucosa of JAM-A^-/-^ mice, supposedly enhancing its vulnerability to acute DSS-induced colitis (42). To address if this increased susceptibility was mediated by the higher number of B and T cells found in the lamina propria of JAM-A^-/-^ mice, JAM-A-deficient mice crossed to recombination activating gene 1 (RAG1) knockout animals lacking B and T cell development were also investigated (43). Lack of adaptive immunity in JAM-A-deficient RAG1^-/-^ mice promoted an even higher susceptibility for development of DSS-induced colitis in comparison with JAM-A^-/-^ animals. However, administration of antibiotics in JAM-A-deficient RAG1^-/-^ mice reduced the susceptibility to disease development. These results suggest an important compensatory role of the adaptive immune system in JAM-A^-/-^ mice that limits bacterial-driven colitis. CD4+ T cells played a relevant role in this compensatory mechanism, as depletion of these cells, but not CD8+ T cells, enhanced susceptibility to colitis. In addition, increased gene and protein expression of transforming growth factor beta 1 (TGF-β1) in colonic tissues pointed to a possible role of this growth factor in the compensatory mechanism. Treatment with anti-TGF-β1 mAb decreased body weight and increased disease activity index scores in JAM-A-deficient mice in comparison to anti-TGF-β1-treated WT mice and to isotype-treated JAM-A^-/-^ mice, demonstrating a protective role of TGF-β1 in JAM-A-deficient mice from developing a more severe acute colonic inflammation.

Mice with selective loss of JAM-A in myelomonocytic cells, progenitors that can differentiate into monocytes, macrophages and subtypes of conventional DCs (44), were used to investigate the role of JAM-A expression on these cells during intestinal inflammation. These mice showed no difference in neutrophil recruitment into the peritoneum and macrophage chemokine production in response to lipopolysaccharides (LPS) or zymosan in comparison with control mice (45). However, these parameters were significantly reduced in global JAM-A^-/-^ mice stimulated by these inflammatory mediators. Mice with selective loss of JAM-A on intestinal epithelial cells demonstrated increased intestinal permeability and reduced peritoneal neutrophil migration and macrophage chemokine production. These findings suggest that JAM-A expression in the epithelium is fundamental for JAM-A -mediated intestinal inflammation.

Exposure of human intestinal epithelial cells to cytokines (TNF-α, IFN-γ, IL-22, or IL-17A) was found to induce JAM-A cytoplasmatic tail tyrosine Y280 phosphorylation (46). Elevated levels of this phosphorylated form were also detected in the colonic mucosa of DSS-induced colitis mice and humans with ulcerative colitis in comparison to healthy mucosa. Further studies will investigate the role of JAM-A cytoplasmatic tail phosphorylation as a regulator of epithelial intestinal barrier functions during bowel inflammation.

### Neurological Disorders

Disruption of the blood-brain barrier of the central nervous system (CNS) leads to leukocyte accumulation in the cerebrospinal fluid, a main component of brain disorders such as meningitis (47) and multiple sclerosis (MS) (48). Surface JAM-A is expressed in brain vessels of MS patients (49) and in vessels of the brain parenchyma and choroid plexus of mice subject to cytokine-induced meningitis (50). In this model of cytokine-induced meningitis, intravenous treatment with anti-JAM-A mAb attenuated meningeal inflammation (50). This therapeutic effect was attributed to inhibition of monocyte and neutrophil accumulation in the cerebrospinal fluid and neutrophil infiltration into the brain parenchyma arising from reduced blood–brain barrier permeability. Further pre-clinical models that aim to discover the effects of JAM-A blockade/promotion on other immunological components involved in neurological disorders may identify JAM-A as a potential target for disease treatment.

### Rheumatoid Arthritis

Rheumatoid arthritis (RA) is a chronic systemic inflammatory autoimmune disease that mainly affects the joints, with an essential participation of the adaptive immune system in its induction phase (51). A breach of self-tolerance led by failure in immune regulatory mechanisms results in activation of immune responses (52) and production of several autoantibodies, against host proteins such as cartilage components, nuclear proteins, stress proteins and citrullinated proteins (53). At the center of these immune responses are leukocytes, that accumulate in the joint, contributing to chronic joint pain (54). Evidence for of JAM-A gene upregulation in human hypoxic cells (55, 56) suggest that the hypoxia present in the synovial tissue of RA patients (57–59) could potentially lead to increased JAM-A gene and protein expression on cells from inflamed joints of these patients. Recently, a study described increased expression of *f11r* mRNA on PMBCs of RA patients (60). Nevertheless, this upregulation could have been driven by the systemic inflammation itself, as healthy individuals were used as control. In addition, JAM-A is expressed in inflamed joints of K/BxN mice (61), animals expressing transgenic T cell receptor (TCR) and major histocompatibility complex class II (MHCII) that develop severe inflammatory arthritis (62).

**Figure 2:**
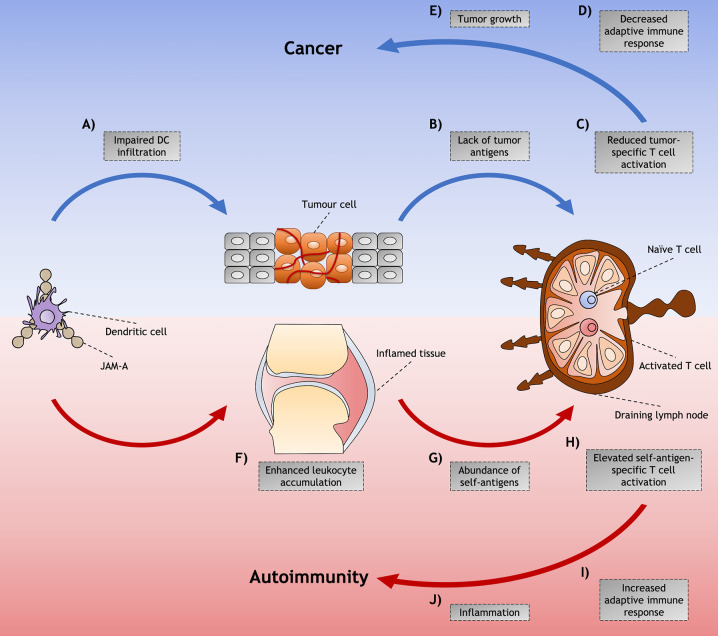
Proposed model for junctional adhesion molecule-A (JAM-A)-mediated dendritic cell (DC) role in cancer and autoimmunity. Although JAM-A may contribute to disease through diverse cell types and signaling processes, mechanisms of DC trafficking mediated by JAM-A and their indirect effects in the immune response might play an important role in the induction and/or promotion of cancer and autoimmunity. Among these is the **(A)** impaired infiltration of DCs that is found in tumors that have achieved the hallmarks of cancer. **(B)** This diminished presence of DCs impairs the availability of tumor antigens that would be presented to tumor-specific T cells in the LNs, **(C)** leading to reduced T cell activation and **(D)** decreased adaptive immune responses against the abnormal cells. **(E)** The lack of immunity against these cells allows tumor to grow and cancer to be set. **(F)** On the other hand, in autoimmunity, JAM-A may assist the accumulation of immune cells in the inflamed tissues by enhancing leukocyte adhesion to the inflamed endothelia. **(G)** The accumulation of DCs increases the availability of self-antigen not only in peripheral tissues - such as arthritic joints of RA patients, where antigen presentation can occur - but also in the draining LN, by DCs carrying self-antigen captured in the affected tissue. **(H)** This abundance of self-antigens possibly leads to the activation of a higher proportion of self-antigen specific T cells. **(I)** The induction of adaptive immune responses against self-antigens leads to **(J)** inflammation and tissue destruction characteristic of autoimmune diseases.

In a model of RA in which autoantibodies from arthritogenic K/BxN drive inflammation and tissue destruction in serum-recipient mice, treatment with anti-JAM-A mAb delayed the disease onset and partially ameliorated overall disease (61). Similar effects were found on mice treated with anti-ICAM-1 mAb, and a more prominent amelioration was found on treatment with mAb targeting the alpha chain of LFA-1, but not the beta chain. ICAM-1 is another ligand for LFA-1 (63), which may suggest that the therapeutic effects in these arthritic mice treated with both anti-JAM-A and anti-ICAM-1 mAbs could have been caused by disruption of LFA-1 pathways. However, more studies are required to investigate the mechanisms involved in JAM-A blockade in models of autoimmune diseases, which may be related to a modulation on immune cells trafficking, as proposed in **Figure 2**.

## JAM-A Manipulation in Cancer Models

The six hallmarks of cancer are sustained proliferative signaling, resistance to cell death, replicative immortality, induction of angiogenesis, evasion of growth suppressors and activation of invasion and metastasis mechanisms (64). Interventions that aim to enhance immune responses to tumor cells, such as increasing tumor-derived antigen presentation to T cells are desirable for cancer treatment. Evidence from the literature suggest that circulating, soluble JAM-A could be used as a biomarker for the detection of some types of cancer, such as multiple myeloma (65) and head and neck squamous cell carcinoma (66). Although few studies show that JAM-A can be downregulated in tumor sites of metastatic breast (67) and anaplastic thyroid carcinoma tissues (68), high levels of JAM-A protein expression in tumor tissues have been correlated with poor prognosis in breast cancer (69) and nasopharyngeal carcinoma (70) patients.

*In vitro* studies using human triple negative breast cancer or thyroid carcinoma cell lines showed increased tumor cell proliferation and migration under JAM-A gene silencing (67, 68). Transfection of a JAM-A plasmid to these cell lines impaired transendothelial migration and colony formation. However, treatment with a JAM-A antagonistic peptide inhibited transmigration of breast cancer cells through inflamed endothelium (71), an important step in the initiation of metastasis formation. Additionally, JAM-A inhibition increased apoptosis and decreased proliferation of multiple myeloma cells *in vitro* and inhibited the progression of this type of cancer *in vivo* (65). In RIP1Tag2 mice, which express the SV40 T-antigen under the rat insulin promoter resulting in carcinogenesis of β cells in pancreatic islets, JAM-A absence decreased growth and aggressiveness of tumors in comparison with control mice (28). An increased tumor specific immune response resulted in diminished angiogenesis and increased apoptosis that was attributed to a more efficient infiltration of JAM-A-deficient DCs, but not macrophages, into tumor sites. This mechanism was dependent on both CD4+ and CD8+ T cells. In a model of multifocal mammary adenocarcinoma, mice lacking JAM-A developed smaller mammary tumors than control mice (72). Disruption of JAM-A pathways increased cell apoptosis and decreased proliferation, however, no differences in angiogenesis or infiltration of DCs or macrophages were found. In addition, treatment with anti-JAM-A mAb suppressed progression of malignant tumors by impairing cell proliferation and angiogenesis in malignant myeloma xenograft murine models (73). Altogether, these studies suggest that although JAM-A expressed by tumor cells may have a protective role on progression of cancer, JAM-A antagonism could enhance immune response against these abnormal cells by possibly facilitating leukocyte infiltration into tumors and DC egress to LNs (see **Figure 2**). Nevertheless, in a recent study, JAM-A-deficient female mice developed a more aggressive phenotype of brain tumor in comparison with WT females and JAM-A–deficient and WT males (74). This study highlights the impact of other factors, such as sex difference, in the therapeutic effects that JAM-A blockade might promote in the development of tumors and demonstrates the complexity and challenges involved in the potential development of a JAM-A-targeted drug for cancer treatment.

## Conclusion and Future Perspectives

Upon recruitment of its extracellular ligands, JAM-A ensures firm adhesion of leukocytes and platelets to the endothelia and plays a definitive role in immune cell transmigration. Antagonistic JAM-A targeting in preclinical models of inflammatory diseases show promising results for controlling inflammation caused by leukocyte or platelet accumulation. In addition, in some cancer models, manipulation of JAM-A pathways achieved agonism of immune responses by affecting leukocyte infiltration into tumors, controlling the progression of the disease. However, the possible disruption in multiple pathways (endothelial cell-endothelial cell, leukocyte-endothelial cell, leukocyte-leukocyte, platelet-endothelial cell, platelet-leukocyte and platelet-platelet interactions) caused by JAM-A targeting suggests precaution in the interpretation of results from preclinical model studies. As such, studies with cell-selective JAM-A disruption that aim to distinguish pathway-specific effects in different pathological conditions will further our understanding of JAM-A role in autoimmunity and cancer and may highlight JAM-A as a potential therapeutic target for human disease.

## Author Contributions

All authors listed have made substantial, direct, and intellectual contribution to the work and approved it for publication.

## Funding

CB was supported by CAPES Foundation, an agency under the Ministry of Education of Brazil (grant number 88881.129556/2016-01). CB, RB, JB, and PG were supported by the Arthritis Research UK (ARUK) programme grant number 19788 and the Innovative Medicines Initiative EU-funded project Be The Cure (BTCURE) [grant number 115142-2]. JB and PG were also supported by the Research into Inflammatory Arthritis Centre Versus Arthritis (RACE) (grant number 22072).

## Conflict of Interest

Author RB was employed by the company Antibody Analytics Ltd.

The remaining authors declare that the research was conducted in the absence of any commercial or financial relationships that could be construed as a potential conflict of interest.
